# London Letter

**Published:** 1894-12

**Authors:** 


### Foreign Correspondence.

### LONDON LETTER.

To the Editor:

Sir,—From time to time there have appeared in the journals references to the recent action of the British Medical Council, whereby the register is, for the present, closed against degrees granted by Michigan and Harvard Universities. Judging by the tone of such references, there must be a considerable feeling of disappointment and indignation; indeed, some writers have worked themselves up to an inordinate degree of righteous wrath. My counsel to all such is, "Spare yourselves, it is only so much lost energy. Do not allow your indignation to run away with you."

The explanation of the recent action of the Medical Council is not far to seek. The great watch-cry of the Republican party yields the answer. Protection! Put yourself, gentle reader, in the place of our English cousins, and, especially if you be a good Republican, you must concur with the dental advisers of the Council. Might not the action of the Council, viewed in this light, be a compliment rather than a slight?

However, I do not write with a view to mollify those indignant spirits who feel their country slighted, but rather to comfort the disappointed ones. There are those who have looked forward to coming out here to practise,—it may be because a brother is already established on this side the Atlantic, or it may be because of a personal knowledge of the field existing here. To all such I would say, "Be of good cheer!" The recent fulminations of the British Medical Council are mere vaporings.

It was the great Irish lawyer, Daniel O'Connell, I believe, who said he could drive a "coach and six" through any act of the British Parliament. Well, in this case it does not even require the genius of an O'Connell. The fact is, the law is hardly worth the paper on which it is printed; any man wishing to practise in the British Isles may come out here and do so with perfect impunity. Let such a one put in addition to his name the name of the school from which he has graduated, and not only can the law not interfere, but no action of any kind whatsoever will be taken by the Medical Council.

The question was recently discussed in an amicable way by the members of a certain dental club here in London. Those who have read the report of the secretary of that club, as it appeared in the *Dental Review*, may remember the allegations of one member, "The Cynic." He charges reputable English dentists with indifference about advertising concerns because they *are publicly known as American.* He has even made the positive statement that it was his opinion that the institutions would long ago have been closed had there not been a tacit understanding among English dentists that it would be of very material advantage to them if the prestige of American dentistry in London could in some way be lowered.

I am not, Mr. Editor, one of the "Mystic Seven," neither do I know "Cynicus." I judge that he is one of those strong, self-reliant characters, intolerant of all sham and crookedness, imbued with a spirit of honesty and lofty aspirations, jealous of the good name of his profession, one the friendly grip of whose hands might well inspire new aspirations and new strivings after excellence. His opinion, no doubt, is based on the following facts: In the provinces the dental law is being enforced, and convictions have followed prosecution. In one case the words used on the sign of the establishment was "Dental Surgery." The offender set up the defence that the words referred not to him, but to the room, and that he had used no words to indicate that he was registered under the act. The courts, however, held, and very properly, that the use of the words was a violation of the act, and convicted the offender. There have been other convictions of a similar character. Why, then, is no action taken in London? Cynicus has given his opinion, and, putting the above facts side by side, it seems justified. The case for Cynicus is still stronger. Among the advertising firms there is one which claims to be distinctively American. One of the leading *directors*, as they love to call themselves, is a man who failed to pass his examinations here, but who returned after a three months' visit to New York, and reported that he had received the D.D.S. while there, and so advertises himself now. Another director was till recently manager of a popular resort of amusement. In the branch over which he presides there is not a single American, and but one qualified man on the premises. The man who is practically manager failed a few years ago to get his degree in the schools here. Yet another director was till recently in a tailoring establishment. In passing, I may say there are many amusing stories told of the efforts of these two directors in interviewing new patients, arranging what work is to be done, and the fee to be paid.

Yet, Mr. Editor, this is an *American* establishment. These are facts known to the whole dental profession in London,—it is not an obscure firm doing business in a small way, but a firm with fine professional (God save the term) premises, sending out their advertisements broadcast. Why are not such charlatans closed up? Is Cynicus right after all? At times I have given a half assent to his proposition. I am convinced, however, that the true cause is deeper. Some time ago we who are registered received a notice from the Medical Council stating that if we permitted an unregistered or unqualified assistant to perform any work of a surgical nature we would be guilty of infamous conduct, and our names would be erased from the register. Now, in the case of the firm alluded to, three directors are "registered," one of the three is an L.D.S., the other two are on the register in virtue of having been in practice before the passing of the act. The remaining directors don't count, as previous to this venture they had no connection whatever with dentistry, one having been manager of a show and the other a tailor or tailor's assistant. Why, then, in the case of the three who are on the register, is not action taken in accordance with the notice which they, in common with the rest of us, must have received? Is it because there is no evidence forthcoming as to a breach of the law on their part? It cannot be so, since their advertisement distinctly says that the patients are operated on by "skilled specialists from the United States," and then gives the names of these men, with the name of the college from which they have graduated. The Council have only to consult their register to find whether these men are legally qualified or not. I take it that in a court of justice it would rest with the advertisers to prove that the men whom they advertise as operators do not operate. But no, Mr. Editor, in the case of this so-called American firm no action is taken, although action based on the said notice has been taken in the case of professedly English dentists. Herein lies the secret of the safety of any American wishing to practise in the British Isles.

Sec. 3 (41 and 42 Vict., cap. xxxiii.) says, "From and after the first day of August, 1879, a person shall not be entitled to take or use the name or title of 'dentist,' . . . or any name, title, or addition, or description, *implying that he is registered under this act.*" The italics are mine. Any man who adds to his name the words "American," or "of Harvard," etc., is exempt, as he does not allege that he is on the British register, he rather wishes it to be understood that he is not, *and he is safe.*

Let me quote two cases in support of this, and which speak volumes. 1st. A dentist is practising not five minutes' walk from the offices of the General Medical Council. He is not on the register. *He* uses his title of "Dr.," and states on his plate at the street door in large letters that he is a D.M.D. of Harvard. Further, he sends out circulars, especially to big dry-goods and fancy warehouses, stating that he is an "ex-demonstrator of Harvard," and will extract teeth gratuitously for the employés if the principals in turn will recommend him to their customers. Here is a case of a man styling himself a dental surgeon, and practising as such within five minutes of the Council, and they cannot interfere with him. He states, however, that he is D.M.D. of Harvard.

Another case: An American doing a legitimate practice, acting and living as a professional man, putting on his name-plate simply the words "Mr. ——," had a call one morning from two young men, whom he afterwards heard were clerks in the offices of the Council. They were so persistent in their desire to know if he was Mr. ——, the dentist, even after he had assured them that if they required dental work he would be glad to examine their mouths, etc., that he finally replied, "No, I am Harry, the horse-thief," and politely ushered them out of the room.

The moral of these two cases is, if you boldly state where your qualification was gained the law cannot interfere. Finally, an

American stated to me that Sir Richard Quain, President of the Medical Council, told him that if he added to his name the words "of New York," no one would interfere with his practising.

I think, Mr. Editor, that I have given sufficient reasons and evidence to prove that any one who wishes can come here and practise without "let or hinderance."

Faithfully yours,

X.

LONDON, ENGLAND, October, 1894.

